# Mapping the Galvanic Corrosion of Three Coupled Metal Alloys Using Coupled Multielectrode Array: Influence of Chloride Ion Concentration

**DOI:** 10.3390/ma11040634

**Published:** 2018-04-20

**Authors:** Hong Ju, JinZhuo Duan, Yuanfeng Yang, Ning Cao, Yan Li

**Affiliations:** 1 College of Mechanical and Electronic Engineering, China University of Petroleum, Qingdao 266580, China; yuangungun_1204@163.com (J.D.); caoning1982@gmail.com (N.C.); yanlee@upc.edu.cn (Y.L.); 2 Corrosion and Protection Centre, The University of Manchester, Manchester M13 9PL, UK; Yuanfeng.Yang@manchester.ac.uk

**Keywords:** desalination, galvanic corrosion, coupled multielectrode array, heterogeneous electrochemistry, chloride ion

## Abstract

The galvanic corrosion behavior of three metal alloys commonly used in water desalination plants was investigated using coupled multielectrode arrays consisting of aluminum-brass (HAl77-2), titanium alloy (TA2), and 316L stainless steel (316L SS). The three electrode types were coupled galvanically and arranged in different geometric configurations. Their corrosion behavior was characterized as a function of the chloride concentration. The potential and current distributions of the three-electrode coupling systems display electrochemical inhomogeneity. Generally, the aluminum-brass wires are anodic versus the titanium alloy and stainless steel. The titanium alloy acts as a primary cathode, and the 316L SS acts as a secondary cathode. The corrosion rate of aluminum-brass depends on the concentration of chloride ion, with a maximum corrosion rate at a chloride concentration of 2.3 wt %. In terms of geometrical arrangements, when the anodic HAl77-2 wires are located on the edge and are connected to the 316L SS wires in the coupling system, the main anodic area enlarges, especially in the area adjacent to the 316L SS wires. When the HAl77-2 wires are located between (in the middle of) the two other types of wires, the corrosion rates are higher than the corrosion rates observed from the other two geometrical arrangements.

## 1. Introduction

According to estimates by the United Nations, nearly 1.8 billion people worldwide will experience severe water scarcity by 2025 [1]. Desalination has been widely adopted in many countries to meet the demand for fresh water [2]. The three most commonly applied desalination technologies are multi-stage flash (MSF), reverse osmosis (RO), and multi-effect distillation (MED) [3]. The evaporators of the MSF and MED technologies are generally constructed with three metal alloys (aluminum-brass, titanium, and stainless steel) and require long-term operation under the aggressive conditions of seawater desalination. Distillation plants are susceptible to the corrosion induced by high chloride concentrations [4], and galvanic corrosion between dissimilar metals is inevitable over time. Although the behavior of two galvanically coupled dissimilar metals is relatively well understood, little effort has been made to study the behavior of more than two galvanically coupled metal alloys.

Generally, conventional macroscopic electrochemical techniques, such as open-circuit potential measurement, potentiodynamic polarization, electrochemical impedance spectroscopy, cyclic voltammetry, and Mott-Schottky measurement, have been applied to study corrosion [5,6,7,8,9]. To achieve spatial resolution during electrochemical measurement, many in situ electrochemical methods have been developed to detect the local defects of corrosion such as the use of coupled multielectrode arrays (CMEAs) [10], also called wire-beam electrodes (WBEs) [11,12,13,14,15,16,17,18,19], scanning vibrating electrode techniques [20,21,22,23,24], scanning electrochemical microscopy (SECM) [23,24,25,26,27], and localized impedance spectroscopy [28,29,30,31].

Based on these microelectrode techniques, the integrated CMEA has been widely used to study the electrochemical inhomogeneity of galvanic corrosion [9,11,18,19]. CMEAs are fabricated from an array of metallic wires that are embedded in an insulating material. Each wire within a CMEA is an individual electrochemical sensor that measures electrochemical parameters and provides a mean value of the electrochemical signal obtained from the local areas of the CMEA surface. It is possible to obtain the average electrochemical signal and the localized electrochemical signals determined by the CMEAs. In addition, CMEAs should consist of an electrode matrix of an equal amount of different metal or alloy wires. Clearly, CMEAs could be ideally suited to investigate the galvanic corrosion of couples consisting of different metals or alloys [18].

Galvanic corrosion usually occurs macroscopically when two or more metals and alloys with different corrosion potentials are immersed within a common corrosive medium. Galvanic corrosion can be affected by many factors, such as temperature, anode-cathode area ratio, and corrosive medium [32,33,34,35,36]. This work investigates the influence of the chloride ion concentration on the corrosion behavior of a three-metal system that is typical of a desalination plant as well as the galvanic effects with three different geometric configurations. 

## 2. Materials and Methods

### 2.1. Electrochemical Setup

All of the electrochemical measurements used here were performed by an automated system, similar to one described previously [11,16,18]. This system consists of a: (a) PXI 1033: a 5-slot PXI chassis with an integrated MXI-Express controller (NI™, Austin, TX, USA); (b) PXI 4071: a 7.5-digit digital multimeter with a voltage measurement range of 10 nV to 1000 V and with an input resistance of more than 1010 Ω (NI™, Austin, TX, USA); (c) PXI 4022: a high-speed, high-precision guard, and current amplifier that can detect picoampere current levels with femtoampere noise with the PXI 4071 (NI™, Austin, TX, USA); and (d) PXI 2535: a high-density FET (NI™, Austin, TX, USA) switch matrix module that features 544 cross-points, 4 × 136 one-wire matrix configuration (136 channels), switching speeds as high as 50,000 cross-points/s, and unlimited simultaneous connection. More channels can be achieved via the facile expansion of switch modules (NI™, Austin, TX, USA). This device is directly controlled by a computer with a transparent, high-speed serial link [18].

### 2.2. Coupled Multielectrode Arrays

**Figure 1:**
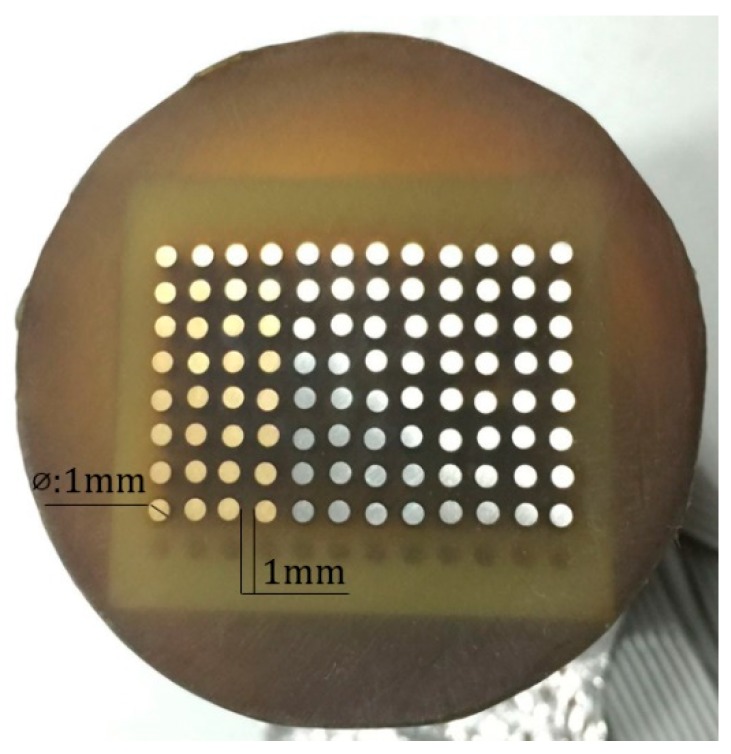
Coupled multielectrode array.

Each CMEA consisted of 96-microelectrodes fabricated from aluminum-brass (HAl77-2), 316L stainless steel (316L SS), and titanium (TA2). As shown in Figure 1 [37], these microelectrodes (1-mm diameter) were arranged regularly within an 8 × 12 matrix and were embedded in epoxy resin with 1-mm spacing in both directions. The total exposed area of the 96 microelectrodes was approximately 0.75 cm^2^. The chemical compositions of real samples (HAl77-2, 316L SS and TA2) are listed in Table 1.

**Figure 2:**
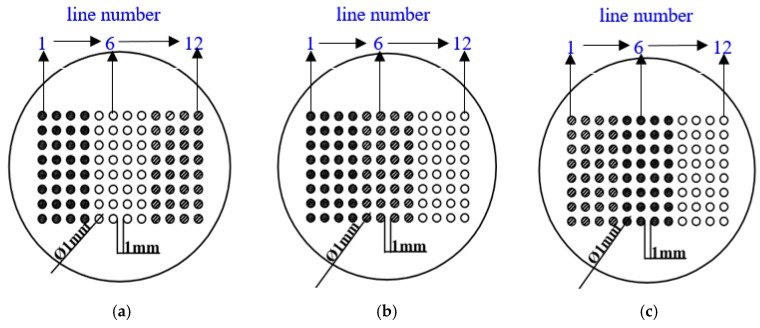
Schematics of three CMEAs with different metal arrangement sequences: (**a**) CMEA1 (HAl77-2/316L SS/TA2); (**b**) CMEA2 (HAl77-2/TA2/316L SS); (**c**) CMEA3 (TA2/HAl77-2/316L SS). (*●* aluminum–brass (HAl77-2), ○ titanium (TA2), ◍ stainless steel (316L SS)).

Three geometric arrangements for the CMEAs were used in this work (Figure 2 [37]). The differences among the arrangements were the relative positions of the three metals. These arrangements were named CMEA1 (HAl77-2/316L SS/TA2), CMEA2 (HAl77-2/TA2/316L SS), and CMEA3 (TA2/HAl77-2/316L SS), respectively. The surface of each specimen was wet abraded using sand papers of 400, 600, 800 and 1000 grade. They were then degreased with acetone and rinsed with deionized water. The CMEAs were stored in a desiccator before testing.

### 2.3. Experimental Conditions

Experiments were performed on the three CMEAs with different geometric metal-arrangement sequences CMEA (CMEA1, CMEA2, CMEA3) in artificial seawater with various concentrations of chloride ions (wt %). The artificial seawater was prepared with ultra-pure water and analytical-grade reagents in accordance with the relevant standard (GB 8650-88). The chloride formulation compositions of artificial seawater with different chloride ion concentrations are shown in Table 2. Open-circuit potential (OCP), local potential, and local current were measured with CMEA technology at 40 °C via the following procedure [18]. First, all wire sensors were disconnected for approximately 30 min to monitor their OCP values. Subsequently, all wire sensors were connected, one by one, within the sequence and remained short-circuited for 12 h. Next, each wire sensor was temporarily disconnected from the other electrodes, its potential was measured, and it was immediately reconnected to the other electrodes. With this approach, the corrosion potential of each wire was recorded sequentially against the saturated calomel electrode (SCE). Currents were measured similarly. The local potential and current measurement processes were controlled automatically by a sequence programmed by a computer developed in an NI™ LabVIEW™ environment. Either the potential or the current measurement was performed within 5 s for all 96 microelectrode wires. Finally, the collected data were transferred to Surfer 8.0 software (Golden Software, Golden, CO, USA) [17] to obtain the local potential and the current distribution maps. Each experiment was repeated by three parallel samples to verify the accuracy of the experiment. The data of three parallel experiments are in good agreement, and the main results are shown as follows.

## 3. Results and Discussion

### 3.1. Corrosion Behavior of CMEA1 in Artificial Seawater with Different Concentrations of Chloride Ions

**Figure 3:**
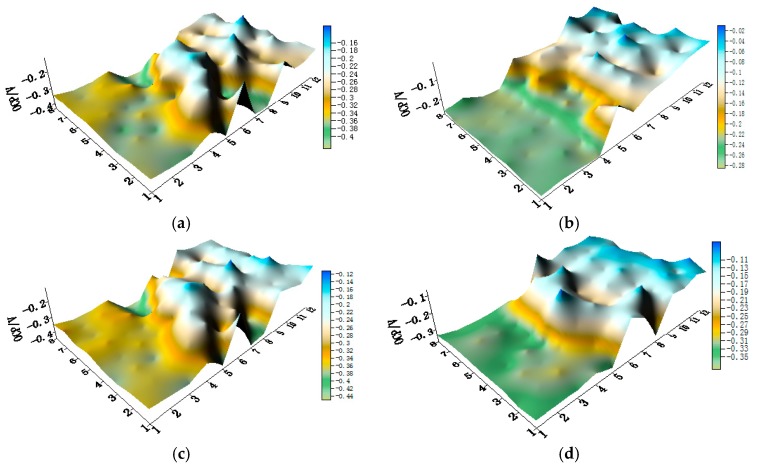

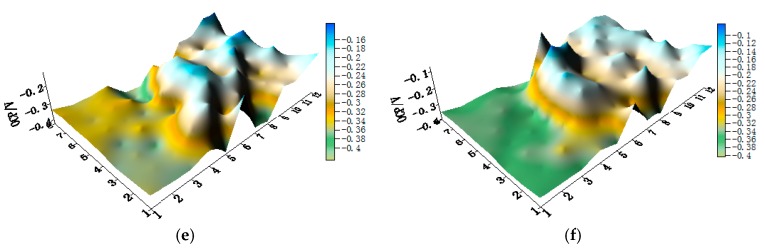
Open circuit potential (OCP) (vs. saturated calomel electrode (SCE)/V) distribution maps of the CMEA1 (HAl77-2/316L SS/TA2) after immersion in artificial seawater with different concentrations of chloride ions (wt %): (**a**) 1.5%; (**b**) 1.7%; (**c**) 1.9%; (**d**) 2.1%; (**e**) 2.3%; and (**f**) 2.5%.

The distribution maps of CMEA1 (HAl77-2/316L SS/TA2 system) at the OCP in artificial seawater with different concentrations of chloride ions are displayed in Figure 3a–f and Figure S1 (supplementary materials). The aluminum-brass (HAl77-2) wires had the lowest potential values of the three metals as a function of chloride ions; the titanium (TA2) had the highest. The OCP value of stainless steel (316L SS) was between HAl77-2 and TA2, but it was closer to TA2. Clearly, HAl77-2 was anodic compared to the other metals, whereas 316L SS and TA2 displayed cathodic behavior and appeared to have less corrosion. In addition, the OCP distribution maps of 316L SS and TA2 were more inhomogeneous than those of HAl77-2, possibly because of the effect of the chloride ions on the passivation films of 316L SS and TA2 in the artificial seawater.

**Figure 4:**
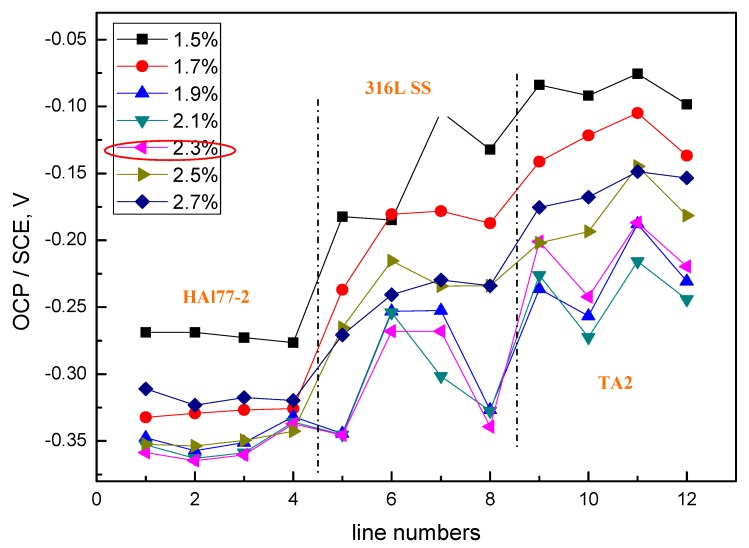
Average potential values of each microelectrode line for CMEA1 at the OCP after immersion in artificial seawater with different concentrations of chloride ions (wt %).

Figure 4 shows the average potential values of each electrode line of CMEA1 at the OCP after immersion in artificial seawater with different concentrations of chloride ions. The results indicate that, for the concentration of chloride ions below 2.3 wt % (heightened), the average potential values of the three metal alloys wires showed an electronegative directional shift with various chloride concentrations, and the potential values became more negative with the increasing concentrations of chloride ions. At the chloride ion concentration of 2.3 wt %, the most negative potential was obtained; afterward, the potential values at the higher chloride concentrations of 2.5 and 2.7 wt % increased. This phenomenon was possibly due to the combined effects of both the chloride ions and the dissolved oxygen concentrations. Specifically, the solution conductivity increased with increasing in chloride ion concentrations—this enhances the effects associated with galvanic coupling among the different metal alloys. However, at high concentrations of chloride, the available dissolved oxygen concentration was reduced, and the cathodic reaction on the materials was restrained. Thus, the corrosion rate dropped. In addition, it was easier to form a scale layer in the artificial seawater with higher salt concentrations, which also contributed to the reduced corrosion rate.

**Figure 5:**
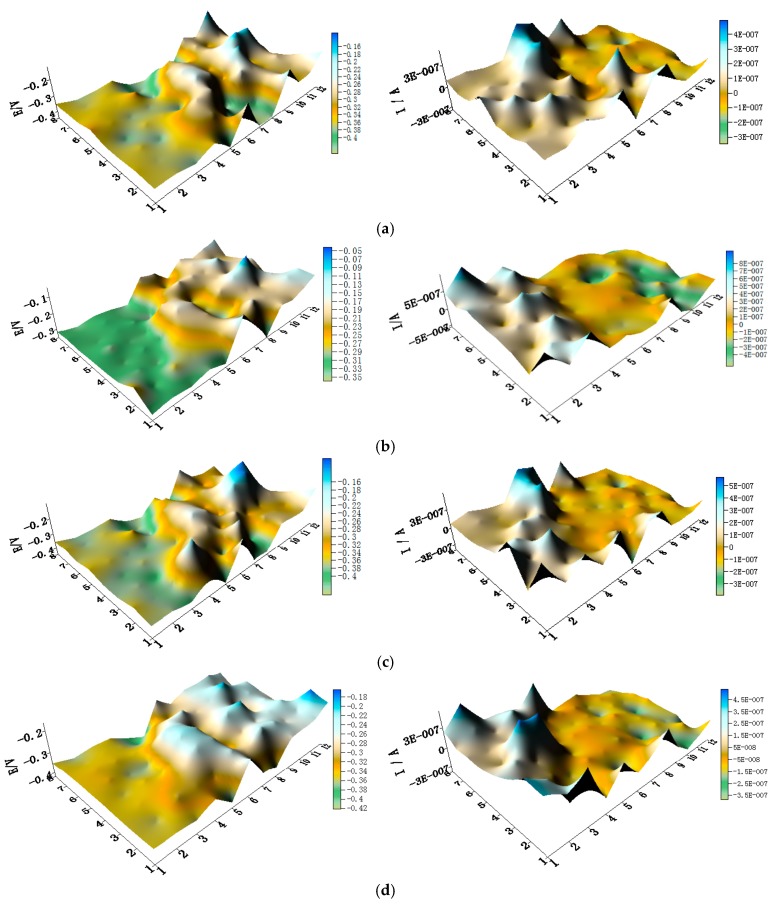

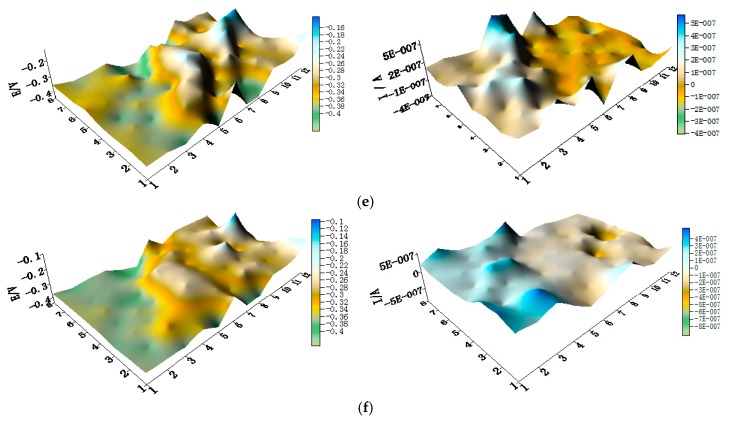
Spatial potential (**left**, V/SCE) and corresponding current (**right**, I/A) distribution maps of CMEA1 after being short-circuited for 12 h in artificial seawater with different concentrations of chloride ions (wt %): (**a**) 1.5%; (**b**) 1.7%; (**c**) 1.9%; (**d**) 2.1%; (**e**) 2.3%; and (**f**) 2.5%.

The potential and the corresponding current distribution maps of CMEA1 after being short-circuited for 12 h in artificial seawater with different concentrations of chloride ions are shown in Figure 5a–f and Figure S2. The local potential of the HAl77-2 wires had the most negative value among the three materials, and its corresponding local currents were the most positive. This implies that the HAl77-2 wires act as an anode in the three-metal coupling system. Also, the potential values of the HAl77-2 wires decreased slightly compared to their initial OCP values (Figure 3). The local potential of the TA2 wires had the highest value in the coupled system, and its corresponding negative local current revealed that the TA2 wires served as the cathode in the three-material coupling system. The local potential values of 316L SS after being short-circuited for 12 h were between the HAl77-2 and the TA2 (the middle)—these values were more negative than their initial OCP values (Figure 3). The negative local currents were apparent on most of the 316L SS wires, and only a few of the 316L SS wires had a positive local current. This finding indicates that most of the 316L SS wires acted as secondary cathodes; only a few of 316L SS wires occasionally acted as a local anode. 

**Figure 6:**
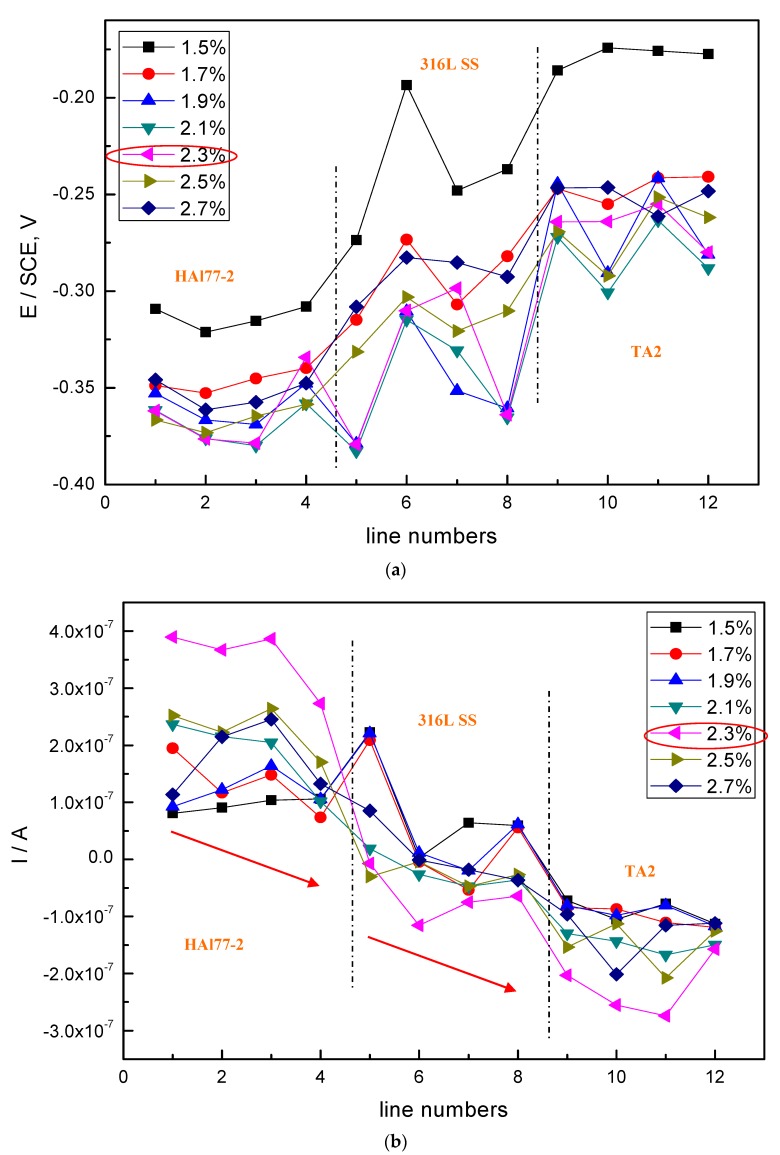
Potential and corresponding current distributions of each CMEA1 microelectrodes line after being short-circuited for 12 h in artificial seawater with different concentrations of chloride ions (wt %): (**a**) average potential; and (**b**) average current.

The average values of the potential and current of each CMEA1 microelectrode line after 12 h in artificial seawater are shown separately in Figure 6a,b. In Figure 6a, the potential values of the HAl77-2 wires initially dropped from line 1 to line 2 and increased slowly along the increasing line number (line 2 to line 4). The potential became more positive with increasing chloride ion concentrations. When the concentration of the chloride ions was 2.3 wt %, the potential of HAl77-2 was the most negative, which might be due to both the galvanic effect and the chloride ions. Meanwhile, in Figure 6b, the local current value of HAl77-2 initially increased and subsequently decreased with increasing chloride concentrations. The maximum current values of HAl77-2 were reached at the chloride concentration of 2.3 wt %. When the current values reached the higher chloride concentrations of 2.5 and 2.7 wt %, the current values dropped again. In summary, the corrosion of the more anodic material is initially accelerated by increasing the chloride concentration in the artificial seawater. However, the corrosion rate starts to decrease when the chloride concentration is higher than 2.3 wt %.

In the CMEA1 system, the 316L SS wires were located between the anode (HAl77-2) and the cathode (TA2). Figure 6a,b show that the fifth-line wire sensors display a lower potential than the other line 316L SS wires (i.e., the sixth to the eighth lines). This indicates indicate a corresponding higher current value than the other line 316L SS wires with the highest chloride concentration. Meanwhile, the potential values of the 316L SS wires from the sixth line to the eighth line increased, and their corresponding current values reduced gradually. Therefore, on the edge condition, the main anodic area enlarged when the anodic HAl77-2 wires were connected to the 316L SS wires in the coupling system. This was especially notable in the adjacent area between the 316L SS and the HAl77-2 wires. 316L SS is not an effective cathode when it is coupled with HAl77-2. Some of the 316L SS wires behaved as local anodes, and the effective anodic area moved from the HAl77-2 wires to the 316L SS wires close to the HAl77-2. 

### 3.2. Corrosion Behavior of CMEA2 in Artificial Seawater with Different Concentrations of Chloride Ions

**Figure 7:**
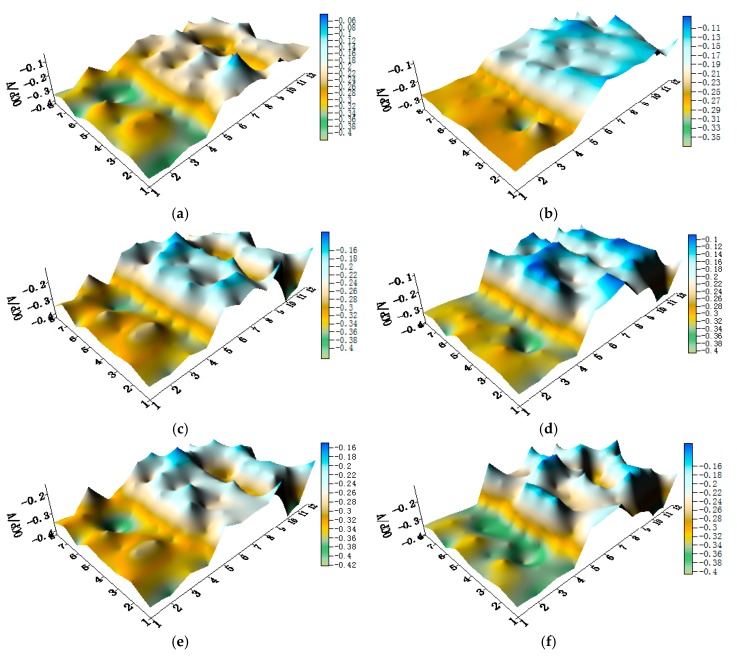
OCP (vs. SCE/V) distribution maps of CMEA2 after immersion in artificial seawater with different concentrations of chloride ions (wt %): (**a**) 1.5%; (**b**) 1.7%; (**c**) 1.9%; (**d**) 2.1%; (**e**) 2.3%; and (**f**) 2.5%.

The OCP distribution maps of CMEA2 (HAl-77-2/TA2/316L SS system) during immersion in artificial seawater with different chloride concentrations are shown in Figure 7a–f and Figure S3. Again, the potential of HAl77-2 among the three materials was the lowest followed by the potentials of 316L SS and TA2, which had the highest potential. The distinct potential between HAl77-2 and TA2 was significantly higher than the difference between 316L SS and TA2. Similar to CMEA1, when the three metals were coupled in the artificial seawater, the HAl77-2 was the anode while 316L SS and TA2 were cathodes. However, the potential distributions on the surface of the HAl77-2 were fairly uniform and the potential values of 316L SS and TA2 were more variable.

**Figure 8:**
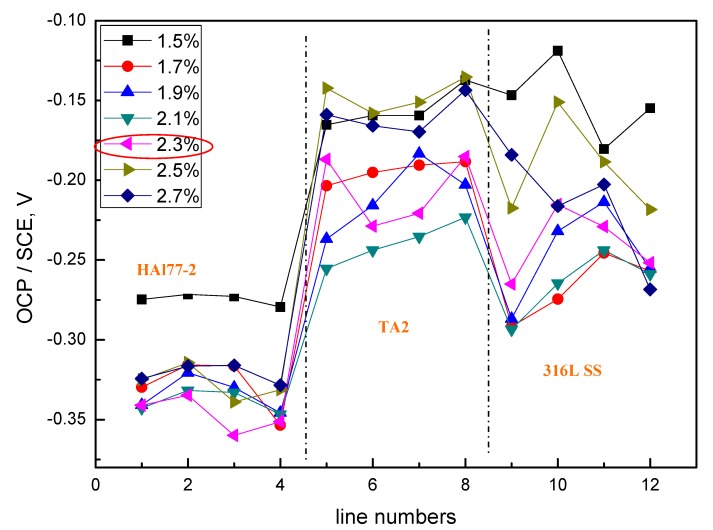
Average OCP potential values of each microelectrode line for CMEA2 after immersion in artificial seawater with different concentrations of chloride ions (wt %).

The average OCP value distributions of each microelectrode line of the CMEA2 immersed in the artificial seawater with different chloride concentrations are shown in Figure 8. The overall behavior, with respect to increasing chloride concentrations, was similar to CMEA1. Significant corrosion was observed at a chloride concentration of 2.3 wt %. At this chloride concentration, the most negative potential was obtained on the HAl-77-2 wires, and a fairly negative potential was observed on the TA2/316L SS wires.

**Figure 9:**
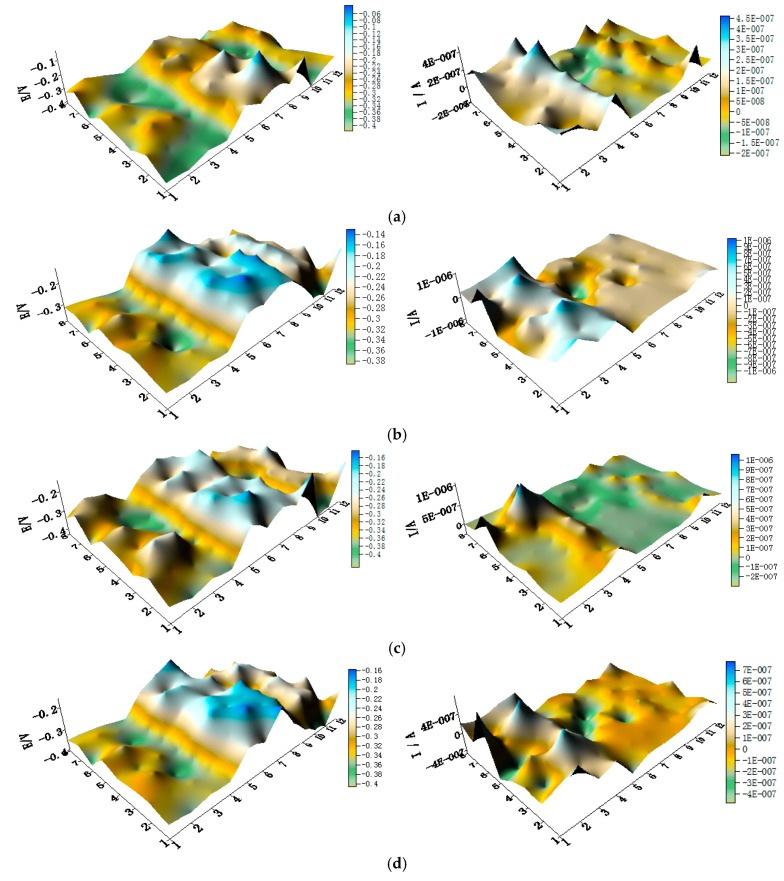

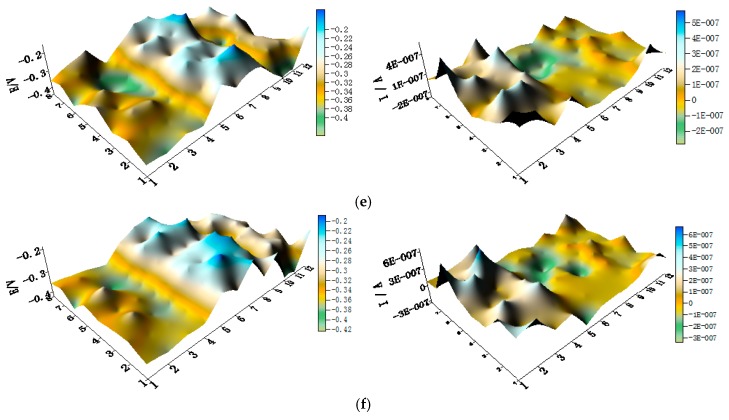
Spatial potential (**left**, V/SCE) and corresponding current (**right**, I/A) distribution maps of CMEA2 after being short-circuited for 12 h in artificial seawater with different concentrations of chloride ions (wt %): (**a**) 1.5%; (**b**) 1.7%; (**c**) 1.9%; (**d**) 2.1%; (**e**) 2.3%; and (**f**) 2.5%.

**Figure 10:**
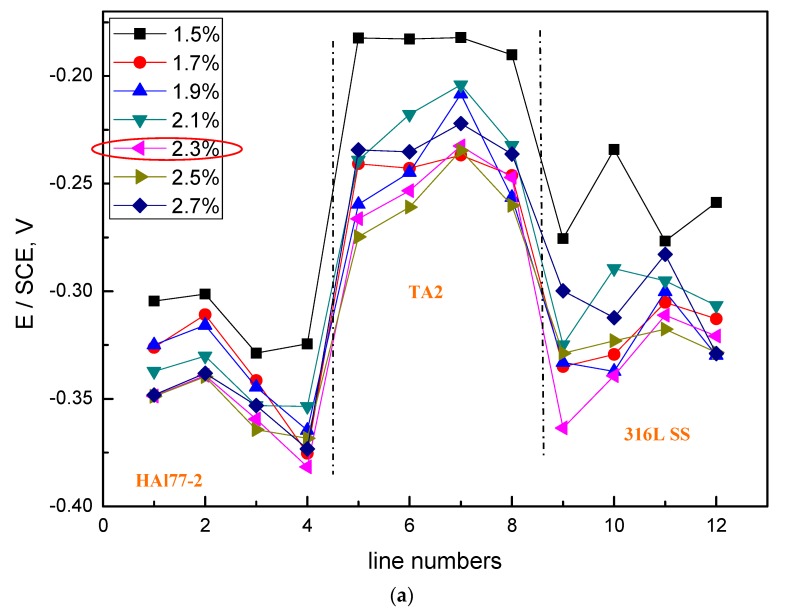

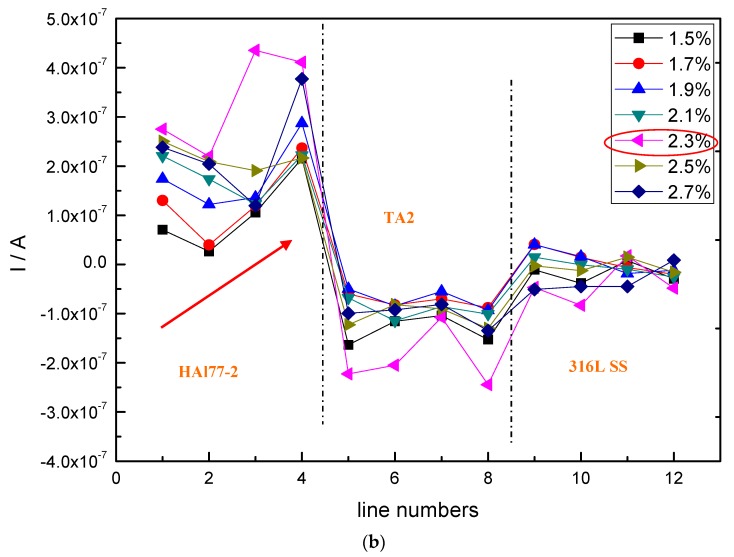
Potential and corresponding current distributions of each CMEA2 microelectrode line after being short-circuited for 12 h in artificial seawater with different chloride concentrations (wt %): (**a**) average potential; and (**b**) average current.

The spatial potential and the corresponding current distribution maps of CMEA2 after being short-circuited for 12 h in the artificial seawater with different concentrations of chloride ions are shown in Figure 9a–f and Figure S4. In addition, the average potential and current distribution of each CMEA2 microelectrode line with different concentrations of chloride ions are displayed in Figure 10. Compared to the initial OCP value shown in Figure 7, Figure 8, Figure 9 and Figure 10 and Figure S4 show more negative potential in the HAl77-2 wire electrode after the connection was short-circuited. Its corresponding local current displayed on the HAl77-2 in the three different wire electrodes was much more positive; thus HAl77-2 served as the corrosion anode in the CMEA2 system. Meanwhile, the local potential value of TA2 was the highest among the three materials, and its corresponding local current was much more negative—indicates that TA2 acted as a cathode in the coupling system.

The potential of 316L SS was between the values obtained by the HAl77-2 and the TA2 but with numerical fluctuations. The current achieved by most of the 316L SS wires was the most negative among the three materials, which indicated that the 316L SS performed mostly as the cathode, even though several currents acquired by the 316L SS wires were positive. This revealed occasional anodic corrosion activity possibly attributable to the damage of passive film on the metal surface. These observations of CMEA2 were similar to the results of CMEA1.

Figure 10 shows the average potential and corresponding current distributions of each CMEA2 microelectrode line after being short-circuited for 12 h in artificial seawater with different chloride concentrations. Similar to CMEA1, the potential of the HAl77-2 (lines 1 and 2) initially shifted to the negative direction and subsequently turned in the positive direction with increasing chloride concentrations (lines 2–4) (Figure 10a). When the chloride concentration was 2.3 wt %, the potential of HAl77-2 was the most negative. Correspondingly, in Figure 10b, the current of HAl77-2 increased first in lines 1 and 2 and then decreased in lines 2–4. Again, the maximum current was achieved at the 2.3 wt % chloride concentration; consequently, the anode corrosion at this point was the most serious. The corrosion behavior trend of CMEA2 was similar to that of CMEA1 in terms of chloride ion concentrations.

Versus the current values shown in Figure 6 and Figure 10, the current distribution fluctuation of the 316L SS wires in CMEA1 was larger than that of the 316L SS wires in CMEA2. The first-line 316L SS wire sensors (the fifth line of CMEA1) for CMEA1 were adjacent to HAl77-2, which displayed an anodic potential and a higher current (Figure 6). Thus, the main anodic area enlarged and even influenced the 316L SS. However, the potential of the 316L SS wires in CMEA2 was higher than that in CMEA1—the current of the 316L SS in CMEA2 was lower than that in CMEA1 because the 316L SS was far from the HAl77-2, which acted as the anode in CMEA2. In addition, the current of the HAl77-2 wires decreased from the first to the fourth line in CMEA1—there was an increase from the first to the fourth line of CMEA2. The fourth line of HAl77-2 in CMEA2 had a higher current value indicating that a more serious and localized corrosion was caused by its direct connection with the cathodic TA2. In CMEA1, a higher anodic current on HAl77-2 was found from line 1–3, and a higher cathodic current was found on TA2 from line 9–11. This was a rather distant current distribution. Similar phenomena appeared in the CMEA3 by a comparison between Figure 6b and Figure 10b, suggesting that TA2 was the most effective cathode with higher cathodic current, regardless of its position.

### 3.3. Corrosion Behavior of CMEA3 in Artificial Seawater with Different Concentrations of Chloride Ions

**Figure 11:**
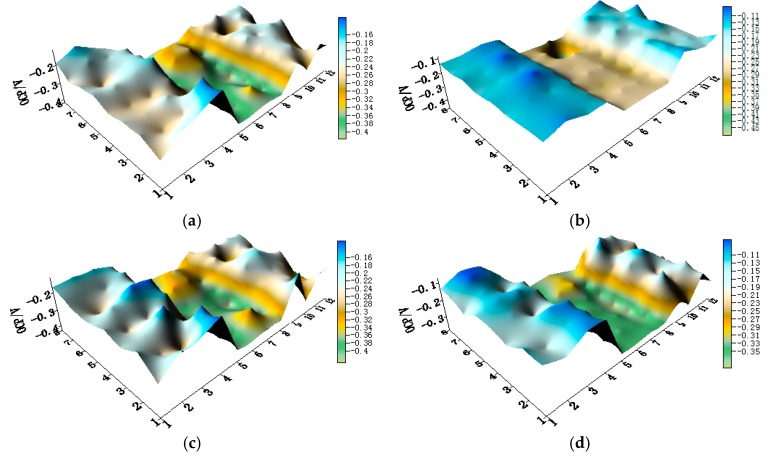

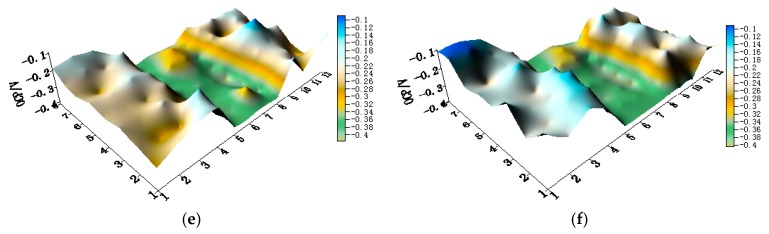
Potential distribution maps of CMEA3 at OCP (V/SCE) after immersion in artificial seawater with different concentrations of chloride ions (wt %): (**a**) 1.5%; (**b**) 1.7%; (**c**) 1.9%; (**d**) 2.1%; (**e**) 2.3%; and (**f**) 2.5%.

The OCP distribution maps of the CMEA3 (TA2/HAl77-2/316L SS) configuration during immersion in artificial seawater with different chloride concentrations are presented in Figure 11a–f and Figure S5. In the CMEA3, the HAl77-2 is located in the central position of the CMEA. As in the previous cases with CMEA1 and CMEA2, the HAl77-2 was anodic with respect to the other two materials. 

**Figure 12:**
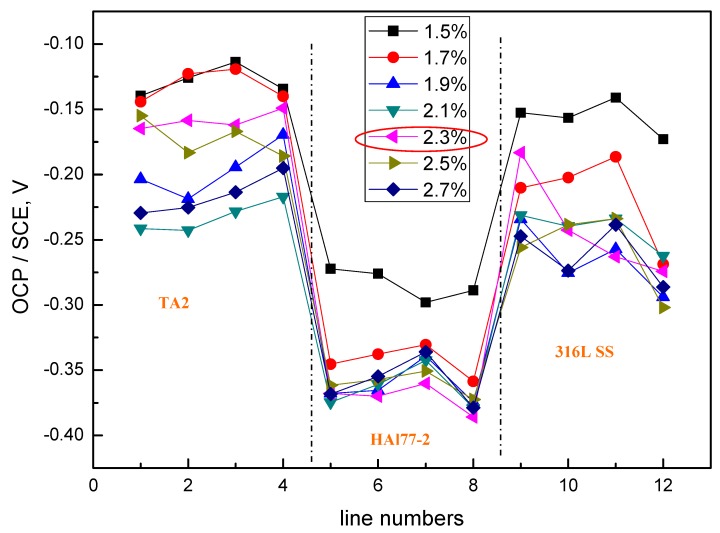
Average OCP distributions of each microelectrode line for CMEA3 after immersion in artificial seawater with different concentrations of chloride ions (wt %).

The average OCP distribution of each microelectrode line for the different chloride concentrations are shown in Figure 12. The potentials of the HAl77-2 wires varied with the different chloride ions concentrations, and the most negative potentials were observed on the HAl77-2 at 2.3 wt %, which coincides with the other two geometric configurations (CMEA1 and CMEA2) shown in Figure 4 and Figure 8, respectively.

**Figure 13:**
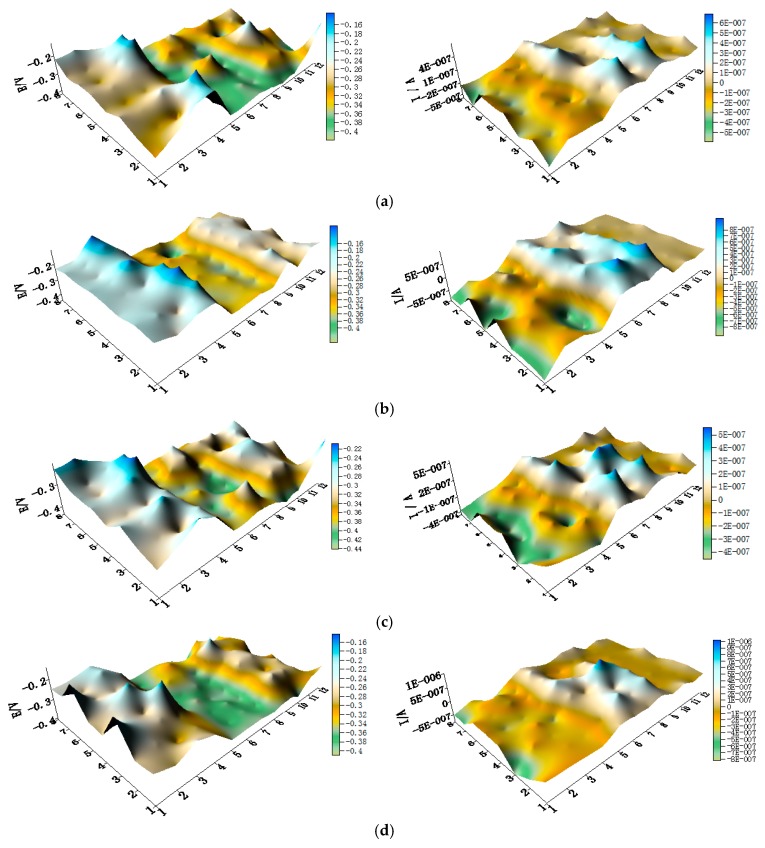

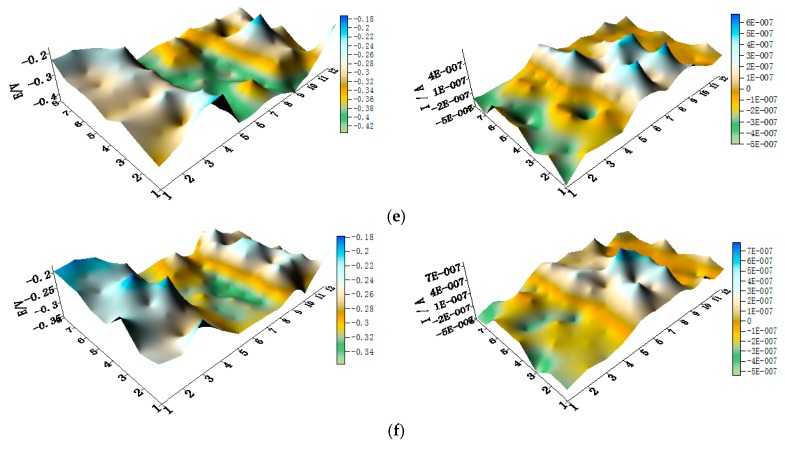
Spatial potential (**left**, V/SCE) and corresponding current (**right**, I/A) distribution maps of CMEA3 after being short-circuited for 12 h in artificial seawater with different concentrations of chloride ions (wt %): (**a**) 1.5%; (**b**) 1.7%; (**c**) 1.9%; (**d**) 2.1%; (**e**) 2.3%; and (**f**) 2.5%.

The spatial potential maps and corresponding current distribution maps of CMEA3 after being short-circuited for 12 h in artificial seawater with different chloride concentrations are presented in Figure 13a–f and Figure S6. The potential maps in Figure 13 and Figure S6 show the local potential of HAl77-2 with the most negative value (middle of maps). Its corresponding local current was positive, indicating that HAl77-2 behaved as the anode in the three-material coupled system. TA2 had the most positive potential value, and its corresponding local current was negative, indicating that TA2 acted as the protected cathode in the coupling system. The potentials of 316L SS were higher than these of HAl77-2, but the potential of 316L SS was lower than TA2.

**Figure 14:**
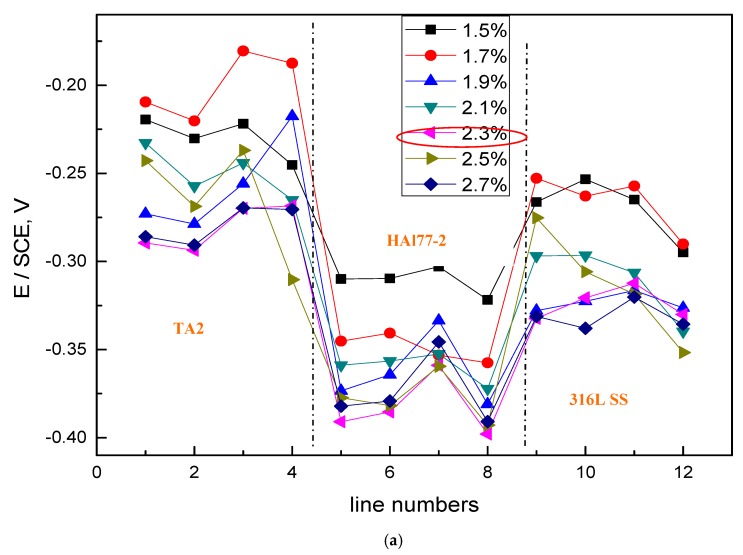

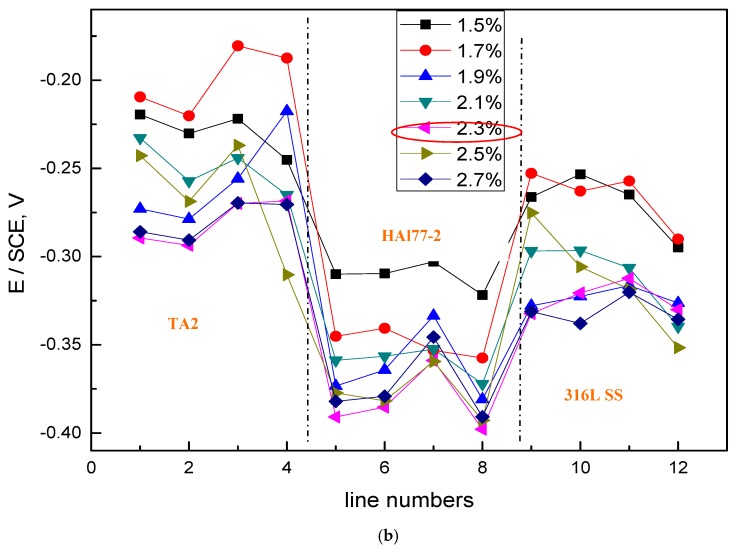
Average potential and corresponding current distributions of each CMEA3 microelectrode line after being short-circuited for 12 h in artificial seawater with different concentrations of chloride ions (wt %): (**a**) average potential; and (**b**) average current.

Figure 14 shows the average potential and the corresponding average current distributions of each CMEA3 microelectrode line after being short-circuited for 12 h in artificial seawater with different chloride concentrations. The currents of HAl77-2 varied with lines 5–8, and higher currents were observed at lines 5 and 8, which adjoin the other two materials, while the lower currents were found in between (line 7). Figure 14b shows the currents at various chloride concentrations at 2.3 wt %, the HAl77-2 currents had their highest values, and the currents of 316L SS and TA2 had their lowest values. This revealed the significant anode behavior on HAl77-2 and the cathode activity on 316L SS and TA2 under these conditions.

Through a comparative analysis of Figure 6, Figure 10 and Figure 14, we see that the potential value of HAl77-2 obtained in CMEA3 was more negative than that achieved in CMEA1 and CMEA2. However, the current of HAl77-2 in CMEA3 was higher than that in CMEA1 and CMEA2. This finding suggests that the corrosion of HAl77-2 is more serious when it is located in the middle of the coupled system. Moreover, the current values of the fifth and eighth lines of CMEA3 were significantly higher than those of the sixth and seventh lines, which suggested that the corrosion at the interface between the anode and the cathode is more serious than away from the interface.

## 4. Conclusions

Since the surface area of each wire in a coupled multielectrode array (CMEA) is much smaller compared to the total electrode working area, each wire surface can be assumed to be electrochemically uniform even if the whole CMEA surface is electrochemically non-uniform. This assumption allows electrochemical theories and methods of describing uniform electroplating and electrodissolution processes to apply to each wire in a CMEA. It is possible to obtain the average electrochemical signal and the localized electrochemical signals determined by the CMEAs. In addition, CMEAs should equally consist of an electrode matrix of different metal or alloy wires. Clearly, CMEAs could be ideally suited for investigating the galvanic corrosion and localized corrosion of different metals or alloys couples.

1. In the three-alloy coupled system, the aluminum-brass (HAl77-2) wires are anodic compared to the titanium alloy (TA2) and 316L stainless steel (316L SS). TA2 acts as a primary cathode, and 316L SS acts as a secondary cathode accompanying the electrochemical inhomogeneity.
2. Regardless of the relative positions of the three materials in the coupled systems, the corrosion of the three-material coupled systems always depends on the chloride concentrations in the artificial seawater—and the corrosion process is highest when the concentration of chloride is 2.3 wt %. When the mass concentration of chloride reaches 2.3%, the local currents of HAl77-2, 316L SS, and TA2 become extreme, and the corrosion of the anodic material is the most serious.
3. The geometrical arrangement impacts the corrosion of the three-material coupled system. In the coupling CMEA1 system, when the anodic HAl77-2 wires are located at the edge and are connected to the 316L SS wires, the current value of the HAl77-2 wires decreases from the first-line wires to the fourth-line wires, and the fifth-line wires (316L SS) display anodic potential and current. As a result, the main anodic area enlarges and is even adjacent to the 316L SS wires near the HAl77-2. In the CMEA2 system, fourth-line (HAl77-2) higher current values are obtained when the anodic HAl77-2 wires are also located on the edge and are directly connected with cathodic TA2—this indicates local anodic corrosion. Furthermore, the CMEA3 system has the most severe corrosion when the anodic material HAl77-2 wires are located between the other two materials (relative to the other two geometrical arrangements).

## Figures and Tables

**Table 1:** Composition of aluminum–brass (HAl77-2), 316L stainless steel (316L SS), and titanium (TA2).

HAl77-2	Composition	Cu	C	Zn							
	wt %	59.33	0.93	39.74							
316LSS	Composition	C	Si	Mn	S	P	Ni	Mo	Cr	Cu	Fe
	wt %	0.02	0.33	1.5	0.02	0.029	10.1	2.1	16.7	0.4	68.80
TA2	Composition	C	Si	Ti							
	wt %	0.26	1.06	98.68							

**Table 2:** Chloride formulation compositions of artificial seawater with different chloride ion concentrations.

	Composition (g/L)	NaCl	MgCl_2_·6H_2_O	CaCl_2_
Cl^−^ (wt %)				
1.5		19.46	8.80	0.92
1.7		21.95	9.94	1.04
1.9		24.53	11.11	1.16
2.1		27.11	12.28	1.28
2.3		29.98	13.52	1.42
2.5		32.59	14.75	1.54
2.7		35.26	16.03	1.67

## Supplementary Materials

The following are available online at http://www.mdpi.com/1996-1944/11/4/634/s1, Figure S1: Open circuit potential OCP (vs. saturated calomel electrode (SCE)/V) distribution maps of the CMEA1 (HAl77-2/316L SS/TA2) after immersion in artificial seawater with 2.7% chloride ions (wt.%); Figure S2: Spatial potential (left, V/SCE) and corresponding current (right, I/A) distribution maps of CMEA1 after being short-circuited for 12 h in artificial seawater with 2.7% chloride ions (wt.%); Figure S3. OCP (vs. SCE/V) distribution maps of CMEA2 after immersion in artificial seawater with 2.7% chloride ions (wt.%); Figure S4. Spatial potential (left, V/SCE) and corresponding current (right, I/A) distribution maps of CMEA2 after being short-circuited for 12 h in artificial seawater with 2.7% chloride ions; Figure S5. Potential distribution maps of CMEA3 at OCP (V/SCE) after immersion in artificial seawater with 2.7% chloride ions (wt.%); Figure S6. Spatial potential (left, V/SCE) and corresponding current (right, I/A) distribution maps of CMEA3 after being short-circuited for 12 h in artificial seawater with 2.7% chloride ions (wt.%).
