# Supplementary material for: Mapping the Galvanic Corrosion of Three Coupled Metal Alloys Using Coupled Multielectrode Array: Influence of Chloride Ion Concentration
Source: Materials (Basel). 2018 Apr 20;11(4):634. doi: 10.3390/ma11040634 (PMC5951518; doi:10.3390/ma11040634)
Supplement: Supplementary file 1 [file materials-11-00634-s001.pdf]

Supplementary Materials

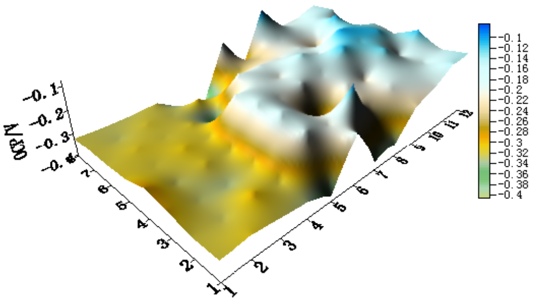

Figure S1. Open circuit potential OCP (vs. saturated calomel electrode (SCE)/V) distribution maps of the CMEA1 (HAl77-2/316L SS/TA2) after immersion in artificial seawater with 2.7% chloride ions (*wt.*%).

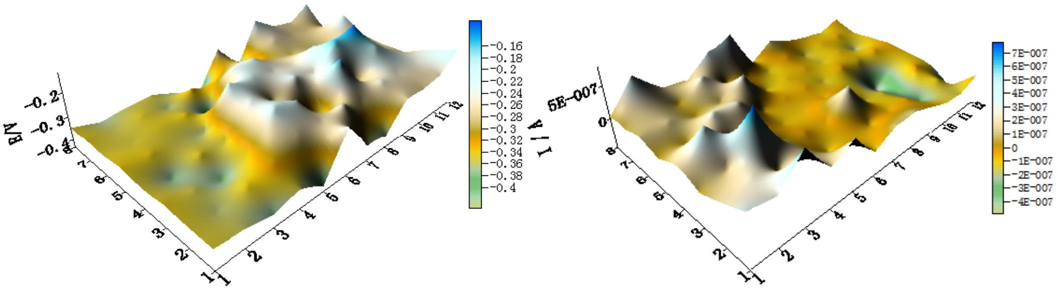

Figure S2. Spatial potential (left, V/SCE) and corresponding current (right, I/A) distribution maps of CMEA1 after being short-circuited for 12 h in artificial seawater with 2.7% chloride ions (*wt.*%).

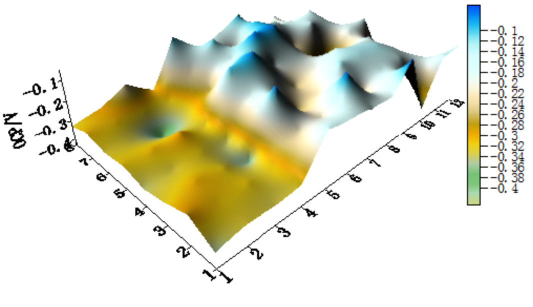

Figure S3. OCP (vs. SCE/V) distribution maps of CMEA2 after immersion in artificial seawater with 2.7% chloride ions (*wt.*%).

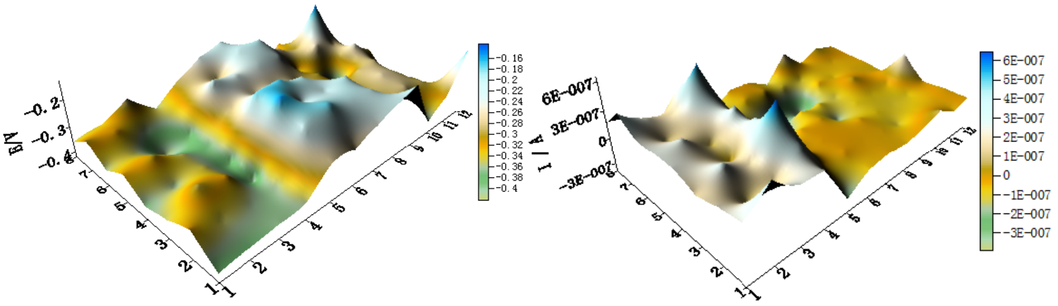

Figure S4. Spatial potential (left, V/SCE) and corresponding current (right, I/A) distribution maps of CMEA2 after being short-circuited for 12 h in artificial seawater with 2.7% chloride ions.

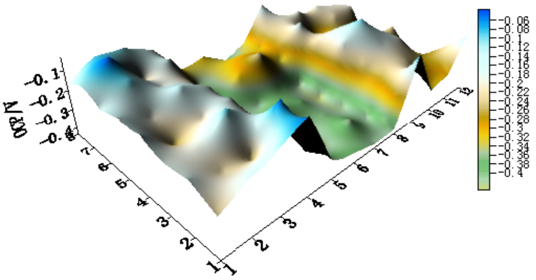

Figure S5. Potential distribution maps of CMEA3 at OCP (V/SCE) after immersion in artificial seawater with 2.7% chloride ions (*wt.*%).

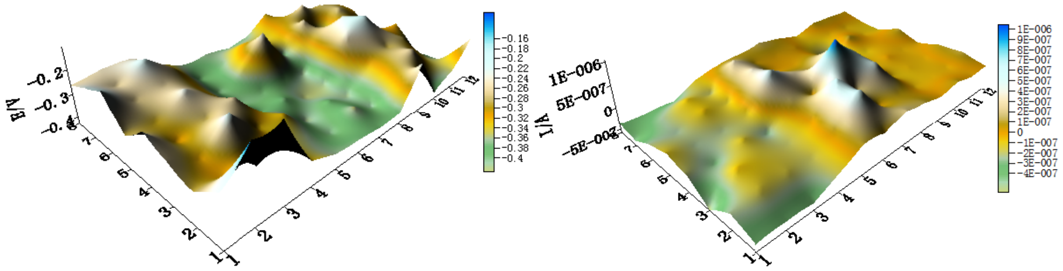

Figure S6. Spatial potential (left, V/SCE) and corresponding current (right, I/A) distribution maps of CMEA3 after being short-circuited for 12 h in artificial seawater with 2.7% chloride ions (*wt.*%).

References

1. Hong Ju; Yuan-Feng Yang; Yun-Fei Liu; Shu-Fa Liu; Jin-Zhuo Duan; and Yan Li. Mapping the Galvanic Corrosion of Three Metals Coupled with a Wire Beam Electrode: The Influence of Temperature and Relative Geometrical Position. *Materials* **2018**, 11, 357, doi:10.3390/ma11030357.

© 2018 by the authors. Submitted for possible open access publication under the terms and conditions of the Creative Commons Attribution (CC BY) license (http://creativecommons.org/licenses/by/4.0/).
